# The Role of Nrf2/sMAF Signalling in Retina Ageing and Retinal Diseases

**DOI:** 10.3390/biomedicines11061512

**Published:** 2023-05-23

**Authors:** Jialing Zhang, Ting Zhang, Shaoxue Zeng, Xinyuan Zhang, Fanfan Zhou, Mark C. Gillies, Ling Zhu

**Affiliations:** 1 Save Sight Institute, Sydney Medical School, Faculty of Medicine and Health, The University of Sydney, Sydney, NSW 2006, Australia; 2 Department of Ocular Fundus Diseases, Beijing Tongren Eye Centre, Tongren Hospital, Capital Medical University, Beijing 100073, China; 3 Faculty of Pharmacy, The University of Sydney, Sydney, NSW 2006, Australia

**Keywords:** Nrf2, sMAFs, antioxidation, retina

## Abstract

Age-related diseases, such as Parkinson’s disease, Alzheimer’s disease, cardiovascular diseases, cancers, and age-related macular disease, have become increasingly prominent as the population ages. Oxygen is essential for living organisms, but it may also cause disease when it is transformed into reactive oxygen species via biological processes in cells. Most of the production of ROS occurs in mitochondrial complexes I and III. The accumulation of ROS in cells causes oxidative stress, which plays a crucial role in human ageing and many diseases. Nuclear factor-erythroid 2-related factor 2 (Nrf2) is a key antioxidant transcription factor that plays a central role in many diseases and ageing in general. It regulates many downstream antioxidative enzymes when cells are exposed to oxidative stress. A basic-region leucine zipper (bZIP) transcription factor, MAF, specifically the small MAF subfamily (sMAFs), forms heterodimers with Nrf2, which bind with Maf-recognition elements (MAREs) in response to oxidative stress. The role of this complex in the human retina remains unclear. This review summarises the current knowledge about Nrf2 and its downstream signalling, especially its cofactor—MAF, in ageing and diseases, with a focus on the retina. Since Nrf2 is the master regulator of redox homeostasis in cells, we hypothesise that targeting Nrf2 is a promising therapeutic approach for many age-related diseases.

## 1. Nrf2 Signalling in Stress and Diseases

### 1.1. Nrf2 Signalling Pathway

**Figure 1:**
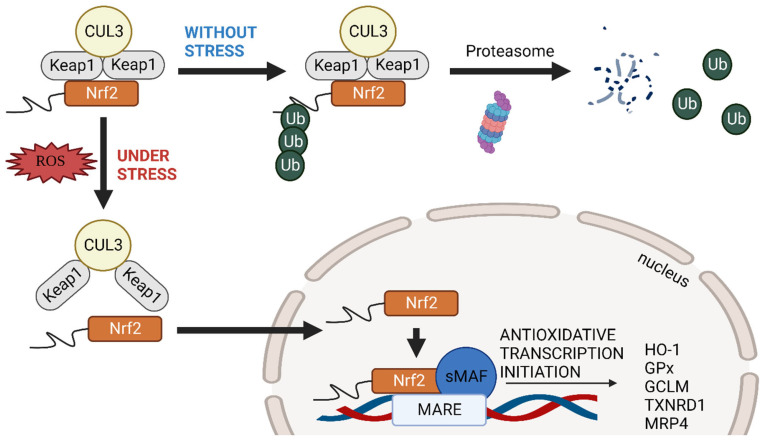
Nrf2 signalling pathway. In the normal condition, the complex of Nrf2–Keap1–CUL3 is degraded via the proteasomal pathway. Upon exposure to stress, Nrf2 is dissociated from the complex and freely moves into the nucleus to form a heterodimer with sMAFs. Upon binding to MARE, it initiates the transcription of antioxidant signalling.

The nuclear factor erythroid 2-related factor 2 (Nrf2) signalling pathway plays an essential role in antioxidation (Figure 1). The key regulatory factors involved in the Nrf2 pathway include Kelch-like ECH-associated protein 1 (Keap1), Cullin 3 (CUL3), and the small MAF subfamily (sMAFs) isoforms. Keap1 can be modified by electrophiles via massive reactive cysteine sulfhydration [1], preventing Nrf2 degradation in stress conditions [2,3]. Without stress, Keap1 binds to Nrf2, for which the complex is degraded via the CUL3-dependent ubiquitination proteasomal pathway [4]. As Nrf2 is unavailable to dimerise with sMAF in the nucleus, the transcription of the downstream antioxidative genes cannot be initiated. sMAFs can homodimerise with themselves and act as transcriptional repressors due to the competition of Maf-recognition elements (MAREs) [5]. sMAFs may also heterodimerise with cap’n’collar (CNC), activator protein 1 (AP-1), and Bach proteins to initiate the downstream transcription of various genes by binding to *MAREs* [5].

Nrf2 is degraded by a protein complex, named the Keap1/CUL3 ubiquitin ligase, under normal conditions (Figure 1) [6]. This degradation process is initiated by adding several ubiquitin molecules (polyubiquitination) to Nrf2, which marks it for destruction by the proteasome. The Keap1 protein acts as a receptor for Nrf2 and helps to bring it into the E3 complex, which is composed of the CUL3 protein and a RING box protein. The RING box protein is responsible for recruiting another set of proteins called ubiquitin-conjugating enzymes (E2), which work together with the E3 complex to transfer the ubiquitin molecules onto Nrf2. Once the E2 enzyme binds to the C-terminal of the CUL3 protein, it catalyses the transfer of the ubiquitin molecules onto Nrf2, leading to its degradation by the proteasome [6].

Under stress (Figure 1), Nrf2 dissociates from the complex of Nrf2–Keap1–CUL3 and moves into the nucleus to bind with sMAFs [2,3,7,8]. Consequently, the heterodimer of Nrf2–sMAFs binds to MAREs and activates the downstream antioxidants, including HO-1 (Heme Oxygenase-1), GCLC (the first rate-limiting enzyme in glutathione synthesis), GCLM (the second rate-limiting enzyme in glutathione synthesis), TXNRD1 (thioredoxin-coded gene), GPx (glutathione peroxidase), and MRP4 (glutathione efflux transporter 4) [7,9,10].

### 1.2. MAF Family

The MAF family contributes to an oncogene and was discovered in the avian musculoaponeurotic fibrosarcoma virus AS42, in the late 1990s [11]. The MAF family is classified into two subfamilies, per their protein size. One is the large MAF isoforms (lMAFs), including MAFA, MAFB, c-MAF, and NRL, which are characterised by the presence of an activation domain at the N-terminal [12]. lMAFs are about 240–340 amino acids in length. The other subfamily is sMAFs, including MAFF, MAFG, and MAFK, which are featured as 150–160 amino acids without activation domains [12].

lMAFs with activation domains independently regulate the transcription of downstream genes. For example, the MAFA and MAFB in beta cells activate the transcription of glucose-sensing genes [13]. Studies suggested that MAFA and MAFB are associated with maturing pancreatic beta cells [14]. In addition, MAFB is also involved in the differentiation of macrophages and monocytes [15] as well as podocyte formation [16]. c-MAF is primarily expressed in the immune system, facilitating interleukin transcription in T cells [17,18]. Moreover, c-MAF also plays a role in liver sinusoidal maturation [19] and lens formation [20]. The physiological role of NRL remains unclear; it specifically expresses in the retina and may be involved in visual function [21].

sMAFs have no activation domains. In stress conditions, they form heterodimers with CNC family members, particularly Nrf2. In the absence of stress, the homodimerisation of sMAFs acts as a transcriptional repressor [22]. sMAFs contribute to the progression of human diseases, such as cancers and cardiovascular diseases. MAFF is abundantly expressed in metastatic breast cancer cells [23]. It regulates the expression of low-density lipoprotein receptors (LDLR) [24]. The expression of LDLR is directly associated with cholesterol metabolism and inflammation, and the dysregulation of these processes leads to CVD [24]. Moreover, induction of MAFF by secretory inflammatory cytokines (IL-1β and TNF-α) can promote host defence against the hepatitis B virus (HBV) [25]. Similarly, MAFK is also highly expressed in triple-negative breast cancer [26]; the upregulation of MAFK influences a tumour’s susceptibility to *salmonella* mucosal infection [27]. MAFK is positively associated with the expression of nuclear factor kappa B (NF-κB) [28], a gene that is activated in cancers, inflammatory responses, and neurodegenerative diseases. The literature indicated that the expression of MAFG is associated with liver cancer [29] and osteosarcoma progression [30]. Overall, all sMAFs form heterodimers with Nrf2 to regulate antioxidative responses [31], which is closely related to the pathogenesis of human diseases.

### 1.3. Nrf2 in Diseases

Nrf2 is a potent transcription factor that controls around 250 genes that are widely involved in antioxidation and detoxification. Nrf2 was found to be involved in oncogenic events in the late 1990s. Tumour cells are susceptible to oxidative stress; the Nrf2–sMAFs complex is overexpressed in tumour cells in response to oxidative stress, to preserve the tumour microenvironment [5]. Mutations of Nrf2 or its repressor Keap1 are also found in cancer cells, resulting in Nrf2 overactivation and promoting cancer cell survival [32]. Nrf2 overactivation provides a satisfactory cancer cell microenvironment, but oxidative stress disfavours tumour growth. In response to oxidative stimuli, tumour cells further upregulate the Nrf2 antioxidative signalling pathway [33,34,35]. This continuous activation status of Nrf2 is closely related to a tumour’s prognosis. For example, the isocitrate dehydrogenase 1 or 2 genes (IDH1/2) of mutated glioma cells have decreased Nrf2 activity but a better prognosis [36]. Nrf2 is also reported to be anticarcinogenic in colorectal cancer and melanoma. Upregulating Nrf2 by activating the PERK/Nrf2/HO-1 pathway induces ferroptosis in colorectal cancer cells [37]. The knockdown of glutaryl-CoA dehydrogenase (GCDH) results in Nrf2 upregulation and promotes cancer cell apoptosis in melanoma [38]. Re-expression of Nrf2, which is deficient in prostate cancer, can protect prostate cells from tumorigenesis [39]. The Nrf2/KEAP1 signalling pathway is also reported to play a critical role in the response of several cancer types to chemotherapy [40,41,42].

In neurodegenerative diseases such as Huntington’s and Hippocampal sclerosis, Nrf2 is low in activity, producing an antioxidative effect. E3 ubiquitin-protein ligase 1 (HACE1) has low expression in Huntington’s, and its depletion decreases Nrf2 expression, leading to neurodegeneration [43]. Nrf2 is downregulated by increasing the activity of its repressor Keap1 in hippocampal sclerosis [44]. Alzheimer’s disease (AD) and Parkinson’s disease (PD) are among the most prevalent age-related neurodegenerative diseases. The oxygen level is usually high in the brain due to the high consumption of coping signals. The continuous accumulation of biological products via lipid peroxidation, DNA oxidation, and free radical production gradually increases local oxidative stress [8]. A Ca^2+^ imbalance due to endoplasmic reticulum (ER) stress and ER-associated ROS production in peripheral blood mononuclear cells is observed in AD patients [45]. The Nrf2-associated antioxidative enzymes show a differential expression pattern in different locations of the brain, such as superoxide dismutase 1 (SOD1), NAD(P)H Quinone Dehydrogenase 1 (NQO1), and HO-1. The Nrf2-associated antioxidative enzymes show a differential expression pattern in different locations of the brain in AD patients, such as superoxide dismutase 1 (SOD1), NAD(P)H Quinone Dehydrogenase 1 (NQO1), and HO-1 [8]. There is also a change in the expression of Nrf2-related antioxidative enzymes in PD patients, with a similar pattern as that in AD patients [8]. Protein and lipid oxidation as well as iron accumulation are also observed in PD, indicating an impaired antioxidative system due to disrupted Nrf2 activity [8]. Sara et al. reported that Nrf2 is highly expressed in PD and is also detectable in leukocytes [46]. Nevertheless, it is recognised that Nrf2 is a potential therapeutic target to be further investigated in both AD and PD.

Nrf2 is also reported to be involved in many other diseases. For instance, Nrf2 is reported to be associated with Hutchinson-Gilford progeria syndrome (HGPS) [47]. This is a rare premature-ageing disease. Its pathogenesis is largely unknown but may result from progerin overproduction [47]. Via a high-throughput siRNA screening, Nrf2 is shown to be a potential risk factor for this disease [47]. In addition, diabetic kidney disease can be improved by β-hydroxybutyrate therapy by inhibiting glycogen synthase kinase-3 beta (GSK3β) and reactivating Nrf2 in glomerular podocytes [48]. An Nrf2 defect is shown in the disrupted homeostasis of ageing and redox system, contributing to fibroblast resistance to apoptosis in diabetic nephropathy [49] and phagocyte ageing and death in human immunity [50].

## 2. Nrf2 Signalling in the Retina

**Figure 2:**
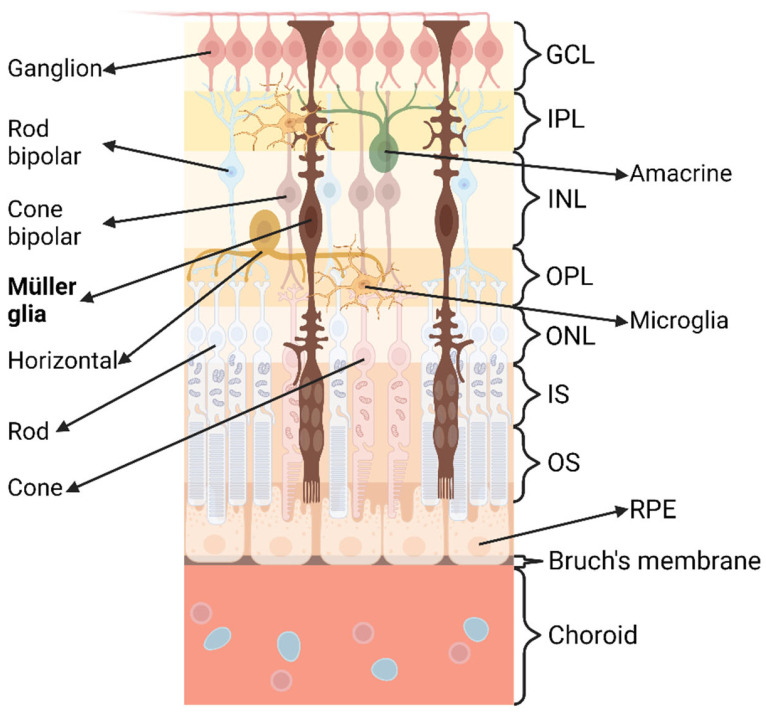
The structure of the human retina. **GCL**: ganglion cell layer; **IPL**: inner plexiform layer; **INL**: inner nuclear layer; **OPL**: outer plexiform layer; **ONL**: outer nuclear layer; **IS**: inner segment; **OS**: outer segment; **RPE**: retinal pigment epithelium.

The retina is a complex, layered structure composed of multiple cell types. In mammals, it consists of five major neuronal cell types and glial cells distributed across five distinct layers (Figure 2). The outermost layer of the retina is the retinal pigment epithelium (RPE), which acts in concert with photoreceptors [51]. Two types of photoreceptors are found in the retina: cones and rods, both of which reside in the outer nuclear layer (ONL). Cones are responsible for daylight due to their lesser light sensitivity than rods [52] and are more concentrated in the fovea, where stronger light passes through [53]. Rods outnumber cones by about 20 fold and are present outside of the fovea [53]. The cell body of the horizontal cell is predominantly situated in the inner nuclear layer (INL) of the retina, and its axon extends into the outer plexiform layer (OPL). Horizontal cells manipulate the synaptic transmission between photoreceptors and bipolar cells [52]. The bipolar cells located in the INL can be divided into cone bipolar and rod bipolar, for their activity in processing information from cone and rod cells, respectively [52]. Amacrine cells are present in the INL and the ganglion cell layer (GCL). The axon of an amacrine cell is projected into the inner plexiform layer (IPL) to form synapses bipolar and ganglion cells [52]. The innermost layer of the retina is the GCL, which is mainly composed of ganglion cells that are the final output neurons to transform visual information from bipolar and amacrine cells to the optic nerve [54].

### 2.1. Nrf2 in Normal Retina

Nrf2 is a key regulator of oxidative stress in the retina. The retina is a metabolically active tissue with high oxygen consumption, which can lead to increased production of ROS. To protect against ROS-induced damage, the retina has an essential antioxidative defence system. Nrf2 plays a central role in regulating this system, particularly in response to stimuli such as ageing, exposure to sunlight, and inflammation. In a normal retina, Nrf2 is expressed in various retinal cell types, including RPE, Müller, and ganglion cells [55,56]. By activating the expression of various antioxidative enzymes, such as HO-1, SOD, and GSH-related enzymes [7,9,10], Nrf2 helps prevent ROS accumulation in the retina and maintain its normal function.

#### 2.1.1. Retina Pigment Epithelium (RPE)

The RPE is located at the outermostof the retina and plays many roles in maintaining the retina structure and the visual cycle. Phagocytotic RPE cells can engulf the dead photoreceptor’s outer segments [57,58]. The RPE also controls the movement of ions, water, and other metabolic wastes out of the subretinal space, as part of the blood–retinal barrier (BRB) [58]. As critical steps involved in the visual cycle, an all-trans-retinal is formed in the photoreceptors; the RPE helps to re-isomerise it to 11-cis-retinal and transport it back to the photoreceptors [58]. Furthermore, the RPE creates a locally immunosuppressive and anti-inflammatory microenvironment essential for the eye’s immune privilege. This is achieved by secreting immunomodulatory cytokines, neuropeptides, and growth factors into the intraocular fluid [59].

The RPE is a protective shield for the retina, while oxidative stress damages the RPE, leading to aged-macular degeneration (AMD) and diabetic retinopathy (DR) [60,61]. The Nrf2 signalling pathway primarily mediates the crucial antioxidative response of the RPE against oxidative stress [56]. Studies report the altered protein expression of Nrf2 and its downstream targets in the RPE. The literature suggests that the protein level of Nrf2 decreases due to its downregulated mRNA expression in the ageing RPE [56]. Similarly, the disruption of Nrf2 signalling is widely found in diseased rodent models and human tissues [56]. A potential upstream regulator (microRNA-144) is positively related to oxidative stress, while the overexpression of microRNA-144 decreases Nrf2 and its downstream targets [62]. As such, it is postulated that Nrf2 may be the master regulator in the RPE to overcome oxidation-induced degeneration. In a recent study, Nrf2 overexpression in mice could successfully rescue the RPE from degeneration [9].

Overall, the RPE protects the human retina from oxidative stress and maintains its redox homeostasis in an Nrf2-dependent manner. The dysregulation of Nrf2 signalling leads to retinal diseases such as AMD and DR. Therefore, Nrf2 is a promising therapeutic target for degenerative retinal diseases. sMAF, as a key regulator of the Nrf2 pathway, is poorly understood in RPE cells, and little is known about its expression in different retinal cell types. However, RPE cells rely on the Nrf2 signalling pathway to protect them from oxidative stress and maintain redox homeostasis. The dysregulation in this pathway leads to retinal diseases such as AMD and DR.

#### 2.1.2. Müller Cells

Müller cells were reported by Heinrich Müller in 1851. They are an essential type of glial cells in the retina, functionally similar to the RPE [63]. They penetrate through whole neuroretina layers. Each Müller cell is responsible for one cone cell [64]. The primate retina has a unique structure, called the macula, at the central posterior. Macular and peripheral Müller cells have distinguished morphologies [64] and transcriptional changes [65]. In mammals, each Müller cell column can contain up to 10 rods, and the arrangement of other cell types in the column depends on the peripheral and macular fovea regions [64]. Müller cells are involved in the inner BRB, which is different from the RPE. Müller cells are responsible for neuroprotection, neovascularisation, and the release of gliotransmitters [63,64]. They are also identified by their stem-like characteristics derived from the retina [66]. The essential physiological activities of Müller cells include maintaining retinal redox homeostasis and supporting neuroretina, due to their extensive distribution across the entire retina [65]. Müller cell dysfunction is associated with many retinal diseases, such as AMD, DR, and macular telangiectasia (MacTel) type 2 [65].

Similarly, Nrf2 also plays an essential role in the stress defence of Müller cells. “Nrf2-mediated oxidative stress response”, including an increase of Nrf2 and the sMAF family, is among the top cellular responses when Müller cells are exposed to strong light (a stress condition) compared to dim light (an unstressed condition) [67]. Nrf2 activation in Müller cells also rescues retinal ganglion cells (RGCs) from cell death [55], since Müller cells support other retinal neurons. In a co-culture model of Müller cells and RGCs, Nrf2 knockout results in a decreased number of RGCs. Nrf2 knockout leads to increasing RGC death and decreasing visual acuity [55]. Furthermore, a novel Nrf2 activator RS9 is reported to protect Müller cell death from light-induced stress [68]. This evidence highlights the crucial role of Müller cells in the retina, while Nrf2 is the key mediator in response to stress.

#### 2.1.3. Photoreceptors

Photoreceptors are composites in the outer retina. Their primary function is to receive light signals and then convert and pass the signal to other neuronal cells by impulses (i.e., horizontal and bipolar cells) [53]. Rods and cones are the two types of photoreceptors that have different physiological characteristics and functions (Table 1). The phototransduction mechanism in rods was fully understood in the early 2000s, while the complex light signal pathway in cones was elucidated later on due to the difficulties of dissecting cones [53]. Cones and rods both rely on glutamate neurotransmission in the synapse, which is formed by horizontal and bipolar cells [53]. Rods have a single synaptic ribbon, whereas cones have many ribbons [53]. The rate of glutamate release in the synapse is primarily influenced by the strength of the light exposure. In the dark, there is a high and rapid release of glutamate, resulting in the photoreceptors hyperpolarising [53].

Photoreceptors also have protective responses against oxidative stress. Mitophagy is a process that degrades the damaged mitochondria to prevent stress and reinstate cellular homeostasis [69]. This mechanism is particularly important in ageing, while impaired mitophagy is largely associated with age-related diseases. Activation of mitophagy in photoreceptors is known to reduce oxidative damage through the Nrf2/p62 signalling pathway [70,71]. p62 has a competitive Nrf2-binding site with Keap1, which is a negative regulator of Nrf2 signalling [72]. By inhibiting Keap1 binding, p62 allows Nrf2 to translocate to the nucleus and initiate the transcription of downstream antioxidative genes, thus maintaining redox homeostasis in the photoreceptors. Overall, Nrf2 drives the mitophagy in photoreceptors to maintain redox homeostasis and protect against oxidative stress.

#### 2.1.4. Bipolar Cells

Bipolar cells mediate the light signal transmission between photoreceptors and ganglion cells. There are various types of bipolar cells, with 13 distinct types in mammals that are classified into two main groups, ON- and OFF-bipolar cells [73]. Rod bipolar cells are a unique type of ON-bipolar cell that specifically receives signals from rods. They depend on amacrine cells as well as ON- and OFF-cone bipolar cells to process these signals and send them as output to ganglion cells [73,74]. Cone bipolar cells are the only type that makes direct contact with ganglion cells [73]. Although rods vastly outnumber cones in mammals, the number of rod bipolar cells is much less than that of cone bipolar cells [75]. This is because every rod pathway requires cone bipolar cells, and rod photoreceptors are 20-fold more than cone photoreceptors in mammals. The primary rod pathway is well-explored in mammals, with signalling transduction occurring through the rod photoreceptors—rod bipolar cells—amacrine cells—ON-cone bipolar cells—OFF-cone bipolar cells—ganglion cells [74,76]. Between the rod photoreceptors and rod bipolar cells, the light signal transduction is mediated by sign-inverting metabotropic glutamate receptor 6 (mGluR6). Then, the signal is passed onto amacrine cells via glutamatergic synapses. Upon receiving the signal, it is distributed to the terminal of the ON-cone bipolar cells via the gap junction and the OFF-cone bipolar cells through the glycinergic synapses [74]. The other two light signal transduction pathways are via cones (cone photoreceptors to cone bipolar cells) or rods that make direct contact with the OFF-cone bipolar cells [76].

Bipolar cells are known to survive after photoreceptor degeneration [77]. However, it is unclear if they are immune to stress. There is limited literature available on the Nrf2-dependent antioxidative signalling in bipolar cells. We suspect that their survival mechanism may not necessarily depend on Nrf2 signalling but rather on peroxisomal β-oxidation, which is crucial for maintaining their retinal functions [78].

#### 2.1.5. Horizontal and Amacrine Cells

Horizontal cells are interneurons located in the inner nuclear layer of the retina that help to transmit signals between groups of cones and rods [79]. The function of horizontal cells depends on their location. The horizontal cell body is in the upper region of the INL, and its axon projects to the cone and rod terminals. At the terminals, the neurotransmitter gamma-aminobutyric acid (GABA) negatively regulates glutamate release [80].

Amacrine cells, like horizontal cells, are associated with the rod and cone pathways. Two types of amacrine cells have been found in mammals, with AII as the primary type. AII amacrines transfer electrical signals to ON-cone bipolar cells and chemical signals (glycine) to OFF-cone bipolar cells, while A17 amacrine cells provide inhibitory feedback (via GABA) to rod bipolar cells [81]. Both horizontal and amacrine cells are rarely associated with age-related diseases; however, natural mutations of the development-related genes in these two cells may result in genetic retinal diseases, such as retinoblastoma [82].

#### 2.1.6. Ganglion Cells

RGCs are the last neuron to transmit visual information to the brain. In humans, a variety of ganglion cells have been identified. The exact clusters of ganglion cells in the human retina are yet to be defined. The two main types of RGCs, ON- and OFF-ganglion cells, respond differently to various light stimuli, such as light frequency and colour [83]. The process of light transduction in ganglion cells is complex. Melanopsin, a photopigment, is expressed in ganglion cells to help with light transduction, even though there are no photoreceptors [84]. As a part of the retina, damaged ganglion cells can impact visual ability.

Oxidative stress predominantly affects ganglion cells and promotes glaucoma [85] as well as other ocular neurodegenerative diseases [86]. Recent studies show that Nrf2 is expressed in RGCs and promotes cell survival under stress. For example, upregulating polo-like kinase 2 (PLK2) through the Nrf2 pathway protects ganglion cells from stress [85]. The literature also reports that Nrf2 upstream factors, including Sox2 overlapping transcript [86], serum response factor [87], and L-carnitine [88], affect the survival of RGCs. Knocking down these inducers leads to RGC loss. Mitochondrial dysfunction is also a primary cause of stress in RGCs, which may lead to glaucoma. RGCs require a high oxygen level and high metabolic activity, resulting in an increased number of mitochondria. The mitochondrial DNA damage is likely associated with ageing and ROS production [89]. The protective function of Nrf2 in mitochondria is widely reported, and Nrf2 activators are shown to support mitochondrial function and structure homeostasis when exposed to stress [71].

#### 2.1.7. MAF Family in Retinal Cells

The exact function of MAF in the retina remains unclear, and the single-cell RNA sequencing data from the Human Protein Atlas (www.humancellatlas.org (accessed on 10 April 2023)) shows that different MAF family members are expressed in different types of retinal cells (Table 2). A human transcriptome dataset better described the expression of metabolic genes to further analyse the expression of the MAF family in the RPE [90]. The MAF family expression pattern in amacrine and retinal ganglion cells is still unclear due to the lack of single-cell RNA sequencing data. The relationship between Nrf2 and sMAF is under investigation, and the mechanism underpinning the activated antioxidant pathways associated with this heterodimer is yet to be fully understood.

### 2.2. Nrf2 in Ageing and Diseased Retina

**Figure 3:**
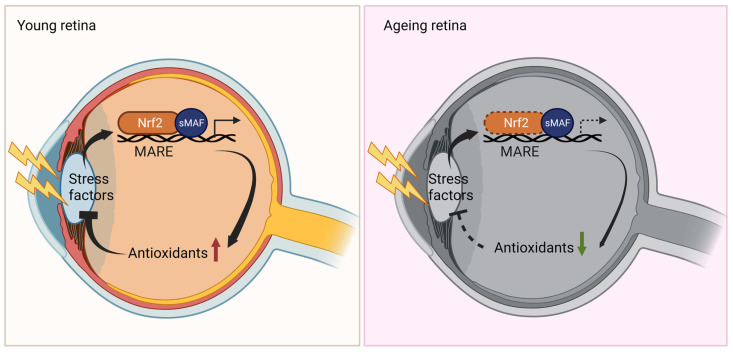
Nrf2 signalling in young and ageing retinas. Nrf2 signalling is initiated in response to stress in the young retina to mitigate the impact of stress factors, such as reactive oxygen species (ROS). This protective effect becomes compromised with ageing in the retina, resulting in a decline in antioxidative responses (indicated by the dashed line).

Research suggests that the disruption of Nrf2 is a significant contributor to age-related and disease-related changes in the retina (Figure 3), including mitochondrial dysfunction and endoplasmic reticulum stress. In human retinal diseases, the activation of Nrf2 decreases, as discussed in the following section. However, currently there is limited knowledge on the role of the MAF family in ageing and retinal diseases.

#### 2.2.1. Dysregulation of Nrf2 in the Ageing Retina—Mitochondria

The human retina is among the most metabolically active tissues in the body. Mitochondria are vital for the normal function of retina cells due to their high oxygen demand. Studies show that in an aged retina, there is a functional decline of the mitochondrial electron transport chain (ETC), leading to the accumulation of ROS that can damage the retina [92]. Mitochondria dysfunction is a key event leading to cell death, which results in retina degeneration. In the aged retina, the number, morphology, and enzymatic activity of the mitochondria are different from those in a healthy retina [93]. Particularly in the RPE cells, the mitochondria are typically regular and round-shaped in younger individuals compared to the larger, more irregular mitochondria found in older subjects [94].

Several mitochondria proteins maintain redox homeostasis, including mitochondria 70 kDa heat shock protein (mtHsp70), mitochondrial uncoupling protein 2 (UCP2), and superoxide dismutase 3 (SOD3). In aged RPE, these proteins have reduced expression [94], which may contribute to the pathogenesis of retina degeneration. Similarly, mitochondria dysfunction in photoreceptors also leads to retina degeneration. Photoreceptors have more mitochondria than the RPE does, predominantly in the inner segment and the axon terminals that are the sites of frequent signalling processing [95]. Oxidative stress is the primary cause of the deletion of photoreceptor mitochondria, further exacerbating the retina-degeneration progression.

Nrf2 responds to ROS accumulation in the mitochondria by mitigating mitochondrial-triggered cell death [96] and shielding against the toxicity and damage induced by the ETC [97,98]. Downstream, Nrf2 activation upregulates the expression of antioxidative genes [99,100,101]. For instance, ferroptosis is a type of cell death triggered by the accumulation of excess iron. The mitochondrial dysfunction in the aged retina leads to disrupted iron metabolism and increased ROS production, which is regulated through the Nrf2 pathway [96]. A study reveals that neuron cells lacking Nrf2 are more susceptible to cytotoxic events, such as the increased calcium resulting from the inhibition of mitochondrial complex I, compared to that of wild-type neurons [97]. The expression of Nrf2 in neurons also facilitates the detoxification of ROS accumulation by inhibiting mitochondrial complex II [98]. These findings suggest that Nrf2 may protect neurons from the toxic events induced by damaged mitochondria. Furthermore, the upregulation of the antioxidant response by Nrf2 may be crucial for protecting retinal cells from oxidative stress.

#### 2.2.2. Dysregulation of Nrf2 in the Ageing Retina—Endoplasmic Reticulum

The endoplasmic reticulum (ER) is the site of protein synthesis and folding. ER stress happens when protein folding reaches its maximum capacity. ER stress also occurs when the retina is exposed to excessive light. The unfolded protein response (UPR) is modulated by stress sensors such as protein kinase RNA-like ER kinase (PERK), inositol-requiring protein 1α (IRE1α), and activating transcription factor 6 (ATF6) [102,103,104]. Suppressed PERK expression in photoreceptors and RPE cells could rescue the retina from light damage [105]. PERK downregulation consistently promotes the expression of the miR-106b-25 cluster to prevent the retina from oxidative stress [106]. The hyperactivation of the IRE1α induced by ER stress contributes to cell death [107]. ATF6 is the major regulator in the ER stress pathway and is associated with cone mitochondrial defects and Müller cell transcriptomic changes [108].

Several studies suggest that the Nrf2 pathway mediates ER stress. Nrf2 overexpression is shown to deactivate the ER stress sensors via the PERK/eIF2α/ATF4/CHOP pathway (i.e., the ER stress pathway), promoting RPE cell survival [109]. This is also evidenced in drug-related studies. The long-term use of minocycline in treating acne vulgaris is toxic to the retina by increasing ER stress and downregulating the Nrf2 pathway [110]. Plant-derived morin hydrate is reported to reduce ER stress in the RPE by activating the Nrf2 pathway [111].

#### 2.2.3. Changes in Nrf2 Signalling in Retinal Diseases

AMD is a common condition, characterised by progressive vision loss, which typically affects individuals over the age of 55 [112]. A clinical hallmark of AMD is the formation of drusen, yellowish extracellular deposits containing lipids and proteins that can be visualised through optical coherence tomography (OCT) [113]. In addition to genetic and environmental factors, excessive ROS production is a known risk factor for AMD [114].

Diabetic retinopathy (DR) is a common complication of diabetes mellitus that is characterised by hyperglycaemia and dysfunction of the blood–retinal barrier (BRB) in the early stage. The high glucose levels in the retina trigger the utilisation of nicotinamide adenine dinucleotide phosphate (NADPH), leading to an increased demand for NADPH and a reduction in the biosynthesis of antioxidants such as GSH [115]. This results in an accumulation of ROS and oxidative stress, accelerating the progression of DR.

The central role of Nrf2 signalling is to maintain redox homeostasis in the retina. These diseases are associated with an imbalanced redox system such as ROS accumulation. In AMD, the expression of Nrf2 is reduced, which may contribute to the imbalanced redox system observed in the disease [56]. In DR, the DNA-binding capacity is reduced, resulting in the low activity of Nrf2 [115]. Furthermore, disruption of the Nrf2 pathway increases the production of the proinflammatory cytokines in relation to the NF-kB pathway [115], contributing to the pathogenesis of DR. More relevant studies about Nrf2 signalling and related retinal diseases are summarised in Table 3.

## 3. Nrf2 Signalling as a Therapeutic Target of Diseases

### 3.1. Manipulate Nrf2 Signalling in Disease Treatments

As previously mentioned, Nrf2 is often activated in cancer cells to promote their survival. Targeting Nrf2 has emerged as a potential strategy for cancer treatment. Several compounds that can suppress Nrf2 activation or promote its degradation are identified, showing improved efficacy in chemotherapies or radiotherapies. For example, brusatol enhances the efficacy of chemotherapy for lung cancer by reducing the Nrf2 level by increasing its degradation [130]. Trigonelline can also promote Nrf2 degradation, improving anticancer treatment [131]. Halofuginone, a derivative of febrifugine, efficiently reduces the Nrf2 protein and shows great potential in treating chemo- and radio-resistant cancers [132]. Ochratoxin A (OTA) is a common food toxin in many European countries, which can decrease Nrf2 by interfering with its DNA binding [133]. ML385 is a small molecule that shows anticancer activity by directly binding with Nrf2 and preventing its activation [134].

Nrf2 activation is considered a viable approach in the therapeutic development of neurodegenerative diseases, especially in treating AD [135] and PD [136]. Sulforaphane, a natural Nrf2 enhancer, is effective in treating epilepsy; however, its clinical applications are largely compromised due to off-target effects and its narrow therapeutic index [44]. In comparison, cyanoenone triterpenoids, with a higher specificity to Nrf2 and wider therapeutic windows, have progressed to clinical trials in epilepsy patients [44]. Withaferin A and amantadine restore the decreased Nrf2 level in dopaminergic neurons by targeting the DJ1–Nrf2–STING pathway, in order to compensate for the neuron loss in PD [137]. In addition, Nrf2 modulators also have potential therapeutic effects on other diseases such as autoimmune, metabolic, respiratory, and CVD [2].

### 3.2. Target Nrf2 Signalling to Treat Retinal Diseases

Dysfunction in Nrf2 signalling is closely associated with certain age-related retinal diseases, such as AMD and DR. Recent studies have attempted to investigate Nrf2-targeted therapies in these diseases. Chrysoeriol is a compound that acts as an Nrf2 inducer, which can increase Nrf2/HO-1 expression to potentially prevent dry AMD [101]. Isothiosynate sulforaphane interacts with the thiols group in Keap1, resulting in conformational changes that cannot suppress Nrf2 activation. This leads to increased expression of Nrf2, which can help resecure RPE and photoreceptors from stress [138]. Triterpenoids are small molecules that may also act on Keap1 to activate Nrf2 and treat AMD. The benefit of these molecules is that they can easily penetrate the BRB [138]. Betulinic acid derivatives protect the RPE and Müller cells via the Nrf2 dependent pathway [139]; lipid nanocarriers may improve the efficacy of compound delivery into these retinal cells [140]. Catapol, an Nrf2 activator, is widely used to treat degenerative diseases and AMD [141]. Chlorogenic acid improves DR resulting from BRB injury by inducing Nrf2 activation [123]. Moreover, herbal extracts/medicines such as ginkgo biloba extracts also demonstrate a cytoprotective effect in the RPE and Müller cells in an Nrf2-dependent manner [142]. Although cell experiments alone may not be sufficient to demonstrate the clinical usefulness of Nrf2 for treating DR and AMD, they do suggest that further research is warranted to determine whether it may have therapeutic potential for these conditions [143].

## 4. Conclusions and Future Studies

Nrf2 is a powerful oxidative defence system widely expressed in cells, which plays a critical role in maintaining cellular homeostasis. The upregulation of Nrf2 in cancers leads to drug resistance in cancer therapies. The downregulation of Nrf2 largely contributes to the pathogenesis of neurodegenerative diseases such as AD, PD, and AMD. Therefore, Nrf2 activity significantly influences the progression of many prevalent human diseases. sMAFs are the key chaperon proteins of Nrf2, which form heterodimers with Nrf2 to regulate downstream antioxidative gene expression. It is unclear whether the loss or low expression of sMAFs is associated with the dysregulation of the Nrf2 pathway in many diseases, especially retinal diseases. Future research is expected to investigate the role of sMAFs and other members of the MAF family in relation to Nrf2 signalling as well as their relationship with human diseases. This information will provide insights into elucidating the mechanism of antioxidative gene regulation and discovering novel therapeutic targets for retinal and age-related diseases.

## Figures and Tables

**Table 1:** Differences between cone and rod photoreceptors.

	Rods	Cones
Shape	Rod-shape in the outer segment	Cone-shape in the outer segment
Distribution	Mainly outside the fovea macular region	Mainly inside the fovea macular region
Numbers	20-fold more than cones	Few
Responsibility	Dim light (sensitive to light)	Daylight (less sensitive to light); colour vision
Cell types	One	Three (L-, M-, or S-type)
Connected cells	Many rods to one bipolar cell	One cone to one bipolar cell
Visual acuity	Less	High
Speed for light response	Slow	Fast
Insufficiency of rhodopsin	Night blindness	Colour blindness

**Table 2:** A summary of MAF family expression in retinal cells from single-cell data [90,91]. ✔ is the symbol for expression; ✔✔ means high expression. **RPE**: retinal pigment epithelium; **PR**: photoreceptors; **BC**: bipolar cells; **AC**: amacrine cells; **RGC**: retinal ganglion cells; **MGC**: Müller glial cells.

	RPE	PR	BC	HC	AC	RGC	MGC
**MAF**	✔✔			✔	N/A	N/A	✔✔
**MAFA**		✔		✔	N/A	N/A	
**MAFB**	✔✔		✔		N/A	N/A	✔✔
**RNL**		✔✔	✔		N/A	N/A	✔
**MAFF**	✔✔				N/A	N/A	✔✔
**MAFK**	✔✔		✔	✔	N/A	N/A	✔✔
**MAFG**		✔	✔	✔	N/A	N/A	✔

**Table 3:** A summary of Nrf2 signalling regulation in retinal disease model.

Disease	Model	Nrf2-Related Signalling and Factors	Conclusion	Ref.
AMD	C57BL/6 mice	Nrf2/SLC7A11/HO-1	HO-1 overexpression inhibition can inhibit RPE ferroptosis and prevent photoreceptor and RPE degeneration.	[116]
DR	ARPE-19 cells	miR-138/Sirt1 */Nrf2	Increased Sirt1/Nrf2 promotes cellular antioxidative response by reducing high glucose-induced ferroptosis-related processes.	[99]
RD *; RP *; AMD	Rd mice	Nrf2-dependent antioxidative response	Activated Nrf2 by plasmid injection to mice reverses the degeneration of RPE cells.	[9]
DR	In vitro (mouse and human retinal microvessels)	LncRNA MALAT1 */Keap1/Nrf2	LncRNA MALAT1 downregulation prevents angiogenesis by regulating Keap1/Nrf2 pathway.	[117]
AMD	ARPE-19 cells	NRF2/IDH * or PPP * Indirectly affecting NADPH expression	Nrf2 is decreased in AMD patients, and NRF2 deficiency leads to cell death and impaired mitochondrial antioxidant response.	[118]
Oxygen-induced retinopathy	C57BL/6J mice	Nrf2/HO-1	Attenuation of retinal neovascularisation is partly affected by the activation of Nrf2/HO-1 signalling.	[119]
DR	MIO-M1 Müller cell; REDD1 knockout B6; 129 mice	REDD1 *-mediated NRF2/GSK3 or Ser-351/Ser-356	Inhibition of REDD1 enhances Nrf2-dependent signalling activity to prevent retinal oxidative stress.	[120]
Dry AMD	C57BL/6 mice	Nrf2/(SOD orGSH-Px or HO-1 or NQO-1)	AP-SD * treatment promotes Nrf2 expression and Nrf2-related downstream gene activities such as SOD, GSH-Px, HO-1, and NQO-1 to reduce the retinal oxidative injury in dry AMD mice.	[121]
Dry AMD	Kunming mice; ARPE-19 cells	Nrf2/HO-1	Activation of Nrf2 signalling protects RPE cells from oxidative stress-induced injury by naringenin.	[122]
DR	C57BL/6 mice; APRE19 cells	Nrf2-dependent antioxidative activity	Chlorogenic acid activates Nrf2 signalling to reduce BRB injury and retinal oxidative injury.	[123]
DR	Wistar rats	Nrf2/xCT *	Elevating Nrf2 expression through system x_c_^−^ increases xCT * expression in DR, which gives a potential treatment for DR progression.	[124]
Non-infectious uveitis	Experimental autoimmune uveoretinitis C57BL/6J mice	Nrf2/HO-1	Nrf2 provides an anti-inflammatory role, while the deficiency of it leads to neuroinflammation in the ocular autoimmunity.	[125]
AMD; DR	Sodium iodate (SI)-induced C57BL/6J mice; ARPE-19 cells	miR-144/Nrf2	Suppression of miR-144 may potentially protect RPE from oxidant-induced damage by activating the Nrf2-dependent redox signalling pathway.	[62]
AMD	ARPE-19 cells	Nrf2/HO-1; NQO-1	Nrf2 activator (luteolin) promotes Nrf2 translocation and, hence, upregulates HO-1 and NQO-1 expression to protect cells against oxidative stress.	[126]
AMD	ARPE-19 cells	Sirt1 */PGC-1α/Nrf2	Natural compound sulforaphane regulates the expression of Sirt1 and PGC-1α * by activating the Nrf2-dependent antioxidative response against blue light, which induces RPE damage.	[127]
DR	Mouse retinal Müller cells; C57BL/6 mice	Nrf2-dependent inflammatory signalling	Natural antioxidant geniposide mediates Nrf2-dependent signalling, leading to the inhibition of ROS accumulation, NF-κB activation, Müller cell activation, and anti-inflammatory response.	[128]
AMD	661w cell line	HMGB2 */Nrf2	HMGB2 is a negative regulator of Nrf2, and its knockdown has been linked to upregulation of Nrf2 signalling, which helps prevent photoreceptor death.	[129]

* Silent information regulator 1 (Sirt1), a protein that belongs to the sirtuin family proteins, has a role in regulating gene expression, DNA repairing, metabolism, and ageing; RD: retinal degeneration; RP: retinitis pigmentosa; long non-coding RNA (LncRNA) metastasis-associated lung adenocarcinoma transcript 1 (MALAT1) plays a role in pre-mRNA splicing; IDH: isocitrate dehydrogenase; PPP: pentose phosphate pathway; regulated in development and DNA damage responses 1 (REDD1), a hypoxia-inducible factor-1 target gene, plays a role in inhibiting the mechanistic target of the rapamycin complex 1 (mTORC1) signalling pathway; solid dispersion of apigenin (AP-SD) is a flavonoid that has antioxidant activity; xCT is a subunit of cystine/glutamate exchanger, mediating the homeostasis of glutamate release and cysteine uptake; PGC-1α: peroxisome proliferator-activated receptor gamma coactivator 1-alpha; HMGB2: high-mobility group protein B2.

## Data Availability

The single-cell RNA sequencing data of MAFF expression in the eyes are from the Human Protein Atlas (https://www.proteinatlas.org (accessed on 10 April 2023)). The RNA data of MAFF expression in the RPE are retrieved from the Database of Genotypes and Phenotypes (dbGaP) (https://www.ncbi.nlm.nih.gov/projects/gap/cgi-bin/study.cgi?study_id=phs001151.v1.p1 (accessed on 10 April 2023)). All data used in this manuscript are available upon the agreement from the lead author.

## Abbreviations

**Abbreviation**	**Meaning**
AC	Amacrine cell
AD	Alzheimer’s disease
AMD	Age-related macular disease
AP-1	Activator protein 1
ATF4	Activating transcription factor 4
ATF6	Activating transcription factor 6
BC	Retinal bipolar cell
BRB	Blood–retinal barrier
bZIP	Basic-region leucine zipper
CHOP	C/EBP-homologous protein
CNC	Cap’n’collar
CUL3	Cullin 3
CVD	Cardiovascular diseases
DJ1	Protein deglycase
DR	Diabetic retinopathy
eIF2α	Eukaryotic initiation factor 2
ER	Endoplasmic reticulum
ETC	Electron transport chain
GABA	Gamma-aminobutyric acid
GCDH	Glutaryl-CoA dehydrogenase
GCL	Ganglion cell layer
GCLC	First rate-limiting enzyme in glutathione synthesis
GCLM	Second rate-limiting enzyme in glutathione synthesis
GSH	Glutathione
GSK3β	Glycogen synthase kinase-3 beta
HACE1	E3 ubiquitin-protein ligase 1
HBV	Hepatitis B virus
HGPS	Hutchinson-Gilford progeria syndrome
HO-1	Heme oxygenase-1
IDH1/2	Isocitrate dehydrogenase 1 or 2 genes
INL	Inner nuclear layer
IPL	Inner plexiform layer
IRE1α	Inositol-requiring protein 1α
IS	Inner segement
Keap1	Kelch-like ECH-associated protein 1
LDLR	Low-density lipoprotein receptor
lMAF	Large MAF
MacTel	Macular telangiectasia
MAF	Avian musculoaponeurotic fibrosarcoma virus
MARE	Maf-recognition elements
MGC	Müller glial cell
MRP4	Glutathione efflux transporter 4
mtHsp70	Mitochondria 70kDa heat shock protein
NADPH	Nicotinamide adenine dinucleotide phosphate
NF-κB	Nuclear factor kappa B
NQO1	NAD(P)H quinone dehydrogenase 1
Nrf2	Nuclear factor-erythroid 2-related factor 2
OCT	Optical coherence tomography
ONL	Outer nuclear layer
OPL	Outer plexiform layer
OS	Outer segement
OTA	Ochratoxin A
PD	Parkinson’s disease
PERK	Protein kinase RNA-like ER kinase
PLK2	Polo-like kinase 2
PR	Photoreceptor
RGC	Retinal ganglion cell
ROS	Reactive oxygen species
RPE	Retinal pigment epithelium
sMAF	Small MAF
SOD1	Superoxide dismutase 1
SOD3	Superoxide dismutase 3
Sox2	SRY-box 2
STING	Stimulator of interferon genes
TXNRD1	Thioredoxin-coded gene
UCP2	Mitochondrial uncoupling protein 2
URP	Unfolded protein response
